# Improving the Stability of Water-in-Oil Emulsions with Medium Internal Phase by the Introduction of Gelatin

**DOI:** 10.3390/foods12152863

**Published:** 2023-07-27

**Authors:** Lei Zhang, Yong Yu

**Affiliations:** 1 Chongqing Science and Technology Bureau, Chongqing 400715, China; kaixinleizi@126.com; 2 College of Food Science, Southwest University, Chongqing 400715, China

**Keywords:** W/O emulsions, stability of W/O emulsions, interface characteristics, gelatin, PGPR

## Abstract

The water-in-oil (W/O) emulsion with a medium aqueous phase may be limited in food and cosmetics due to its poor stability and high cost. Herein, this work proposed a facile strategy to improve the W/O emulsion stability by introducing gelatin. The influence of different gelatin concentrations (0, 0.5%, 1.0%, 2.0%, and 4.0%) on the stability and properties of W/O emulsions was mainly investigated. Results showed that the obtained emulsions still belonged to W/O emulsions after adding gelatin to the aqueous phase. As the gelatin concentration increased (0~4.0%), the interfacial tension decreased, which is conducive to promoting the interface adsorption of polyglycerol polyricinoleate (PGPR). Furthermore, introducing gelatin also improved the water-holding capacity (WHC) (33.50~6.32%) and viscosity of W/O emulsions and reduced the droplet size (37.47~8.75 μm) of emulsions. The enhanced interfacial adsorption and aqueous gelation induced by gelatin addition promoted the formation of a tight overall emulsion network structure by the interaction between the interfacial adsorbed PGPR, as well as PGPR and gelatin in the aqueous phase. The enhancement of the overall network effectively improved the storage stability (35 d), thermal stability (20 min, 80 °C), and freeze–thaw stability (10 cycles) of emulsions, especially at 4.0% gelatin concentration. Hence, this study can provide guidance for the improvement and regulation of the stabilities of W/O emulsions.

## 1. Introduction

The emulsion is a colloidal dispersion formed by two kinds of immiscible liquid. It can not only realize the loading and controlled release of active ingredients but also realize the precise customization of materials, which has gradually become a research hotspot and is widely used in food, agriculture, medicine, materials, and other fields [1,2,3,4]. Generally, the emulsion is mainly divided into single-layer emulsions and multi-layer emulsions. The single-layer emulsion mainly includes oil-in-water (O/W) and water-in-oil (W/O), and the multi-layer emulsions mainly include oil-in-water-in-oil (O/W/O) and water-in-oil-in-water (W/O/W) [5,6,7]. Compared with multi-layer emulsions with strong thermodynamic instability, the single-layer emulsion is more likely to attract the research interest of many scholars. It may also be more suitable for industrial production and application because of its simple preparation and strong stability. For single-layer emulsions, O/W emulsions can prevent lipid oxidation, replace fat, protect fat-soluble ingredients, personalized 3D printing, etc., which is favored by many experts and scholars [8,9]. For example, Liu et al. [10] applied cod protein-stabilized emulsions to fish cake instead of fat. Research showed that adding emulsions can upgrade the distribution of water or oil in fish cake and promote the formation of a tight gel network to better retain water and oil, thus producing softer and healthier fish cake. Zhang et al. [11] used sea bass protein microgel to prepare ultra-stable high internal emulsions. The emulsion showed excellent thixotropy and creep, 3D printing performance. Furthermore, the produced emulsion improved the physicochemical stability and bioavailability of astaxanthin.

However, there are relatively few studies on the application of W/O emulsions at present. On the one hand, the W/O emulsion is prone to oil-water separation and fat floating, showing poor stability, which severely limits the large-scale application of W/O emulsions in related fields. On the other hand, the content of the aqueous phase in W/O emulsions is relatively low (≤30%, *v*/*v*), which may limit the high load of water-soluble functional components. For instance, Gao et al. [12] used beeswax to prepare W/O emulsions with a 10–55% (*v*/*v*) aqueous phase and found that the emulsion had obvious aqueous phase precipitation, fat floating, and other instability phenomena after storage for 30 d. Thus, it is a severe challenge to manufacture stable W/O emulsions with high aqueous phase content. In addition, excessive consumption of edible oil is undoubtedly detrimental to health. By increasing the water phase content, the W/O emulsion can obviously reduce fat content and further reduce health risks, which can also increase the load capacity of water-soluble functional components and enrich their nutrition or sensory properties [7]. Therefore, increasing the aqueous phase content and improving the stability of W/O emulsions has a very important application prospect for the food and cosmetics industry.

In recent years, the aqueous gelation induced by adding a polymer may be an effective strategy to improve the stability of emulsions, which has been successfully applied in multi-layer emulsions (PGPR ≥ 4.0%) [13,14]. It mainly enhances the interaction between interfacial particles and the polymer in the water phase to enhance interfacial stability, thereby inhibiting the precipitation of water droplets and the upward floating of oil droplets [15,16]. Thus, it is theoretically feasible and simple to improve W/O emulsions stability through aqueous gelation. According to the current reports, the common gel-forming agent mainly includes protein (i.e., whey protein, pea protein), polysaccharide (i.e., xanthan gum, arabic gum, methyl cellulose, starch), or their complexes [17,18,19]. Interestingly, gelatin from animal by-products is considered one of the most common food additives due to its advantages of wide source, low cost, easy availability, and high safety. For example, gelatin can be used as a gel, emulsifier, film-forming agent, and clarifying agent [20,21]. Compared with the above gel-forming agent, gelatin with good gelling properties and high safety may be more suitable for inducing aqueous gelation [22]. In addition, to our knowledge, there is no research on adding gelatin to induce aqueous gelation so as to improve the aqueous content and stability of W/O emulsions.

Therefore, in current research, the polyglycerol polyricinoleate (PGPR, 5.0%, *w*/*w*) and gelatin were used as lipophilic emulsifiers and gelling agents, respectively, and the effects of different gelatin concentrations (0, 0.5%, 1.0%, 2.0%, and 4.0% *w*/*v*) on the visual appearance, water-holding capacity (WHC), interfacial tension, microstructure, droplet size, apparent viscosity, storage stability, thermal stability, and freeze–thaw stability of the W/O emulsion with medium aqueous phase (50%) were mainly investigated. The purpose of this work is to provide a strategy for improving and adjusting the W/O emulsion stability.

## 2. Materials and Methods

### 2.1. Materials

Polyglycerol polyricinoleate (PGPR) was obtained from Shanghai Maclean’s Biochemical Technology Co., Ltd. (Shanghai, China). The total content of dimer, trimer, and tetrameric glycerol in PGPR is ≥75%, and the content of heptaglycerol and above is ≤10%. Sigma-Aldrich (St. Louis, MO, USA) provided gelatin (type A). Jiusan Grain and Oil Industry Group Co., Ltd. (Harbin, China) offered commercial soybean oil. All chemicals or solvents used in this work were of analytical grade and used directly without further purification.

### 2.2. Preparation of Emulsions

The 5.0% PGPR was added to soybean oil, heated in a 55 °C water bath, and stirred (HH-4 magnetic stirrer water bath pot, Changzhou Guohua Electrical Appliance Co., Ltd., Changzhou, China) for 15 min to obtain the oil phase. The different qualities of gelatin are then accurately weighed and added to pure water, heated, and stirred in a water bath at 60 °C for 10 min to prepare gelatin solutions of different concentrations (0, 0.5%, 1.0%, 2.0%, 4.0% *w*/*v*) to act as the water phase. Finally, the oil phase was added to the glass bottle (20 mL, d = 27.5 mm, h = 57 mm) containing the water phase in a ratio of 1:1 (*v*/*v*), and the emulsion (10 mL) could be formed by high-speed shear (T18 homogenizer, IKA Works, Inc., Wilmington, NC, USA) under certain conditions (15,000 r/min, 90 s).

### 2.3. Appearance Observation of Emulsions

The phone camera was used to record the visual appearance of emulsions.

### 2.4. Emulsions Type Test

The droplet type of emulsion was tested by referring to the method of Feng et al. [23]. In simple terms, 100 μL of emulsions was added to 50 mL of pure water, and the emulsion type was determined by observing the dispersion behavior of the droplets in the water. When the emulsion floats on pure water, the emulsion type is W/O; otherwise, it is O/W.

### 2.5. WHC Test

The WHC was tested by the ratio of water layer height to the total height of emulsions to evaluate the ability of emulsions to load water.

### 2.6. Interfacial Tension Test

The interface tension was measured referring to the previous method using a Sigma 700 interface tensiometer (Boerlin Technology Co., Ltd., Gothenburg, Sweden) [22]. Briefly, 45 mL of soybean oil containing 5% PGPR was first used to calculate the correction compensation, and then 45 mL of gelatin solutions with different concentrations (0, 0.5%, 1.0%, 2.0%, 4.0% *w*/*v*) were added to the sample cell. According to the prompt, 45 mL soybean oil containing 5% PGPR was slowly added to it. Furthermore, the speed down, speed up, wetting depth, measurement time, sample interval, stabilize, integrate, detect range, start position, reset speed, and temperature were 20 mm/min, 20 mm/min, 12 mm, 60 min, 1 s, 4 s, 4 s, 2 mN/m, 5 mm, 40 mm/min, and 25 °C, respectively. The interface tension number was directly provided by the testing software (Oneattension, 2019).

### 2.7. Microscopic and Droplet Size Test

The sample (5 μL) was placed on a clean glass slide and covered with a cover glass. Then, the microstructure of emulsions was measured using a microscopy equipped with 20× objectives (BX53, OLYMPUS, Tokyo, Japan). Based on the microscopic image, 300 droplets on the picture were randomly selected, and the droplet size of the sample was calculated by using the Nano Measure software (V1.2).

### 2.8. Viscosity Test

The viscosity of emulsions could be gained by using an MCR302 rheometer (ANTON PAAR GMBH, Australian) loaded with a conical plate (50 mm). Shear scanning was performed at a height of 1 mm from the sample to the conical plate at a frequency of 0.1 to 100 s^−1^ (25 °C) [24].

### 2.9. Emulsions Stability Test

#### 2.9.1. Storage Stability

The emulsion was placed in a refrigerator at 4 °C to investigate its storage stability through visual appearance, microstructure, and droplet size.

#### 2.9.2. Thermal Stability

The emulsion was heated at 80 °C for 20 min to investigate its thermal stability through visual appearance, microstructure, and droplet size.

#### 2.9.3. Freeze–Thaw Stability

The emulsion was first put in the refrigerator (−20 °C) for 24 h and then put in a 25 °C water bath for 2 h, which is a freezing and thawing cycle. The freeze–thaw stability was investigated by visual appearance before and after freeze–thaw.

### 2.10. Statistical Analysis

All experiments were carried out at least triplicate independently at 25 °C. The obtained data were presented as mean values ± standard deviation (SD). The one-way analysis of variance (ANOVA, *p* < 0.05) and Duncan’s multiple range test were performed using SPSS software (version 17.0) and Origin 8.6. A value of *p* < 0.05 was considered to be statistically significant.

## 3. Results and Discussion

### 3.1. Analysis of Appearance and WHC

**Figure 1:**
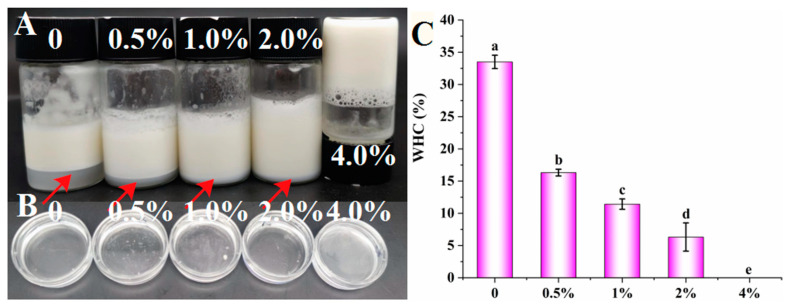
The appearance (**A**), dispersed behavior (**B**), and WHC (**C**) of emulsions stabilized by PGPR and gelatin (0, 0.5%, 1.0%, 2.0%, and 4.0%). Different lowercase letters (a–e) indicate significant differences (*p* < 0.05).

The effect of different concentrations of gelatin solution (0, 0.5%, 1.0%, 2.0%, and 4.0%) on the appearance and WHC of emulsions is displayed in Figure 1A,C. All samples could form milky white emulsions due to the good emulsification performance of PGPR or gelatin. PGPR belongs to a lipophilic emulsifier, which easily forms W/O emulsions [25]. In this system, gelatin was introduced to induce aqueous gelation. However, gelatin is a hydrophilic emulsifier, which is easy to form O/W emulsions [22]. Hence, in order to determine the emulsion type, the droplet type testing was conducted, and the results are shown in Figure 1B. All emulsions floated on the water indicated that all the prepared emulsions were W/O emulsions, which is consistent with the report by Cheng et al. [26]. As can be observed from Figure 1A, with the gelatin concentration increased from 0 to 2.0%, the water layer gradually decreased, and the WHC decreased significantly (*p* < 0.05) from 33.50% to 6.32% (Figure 1C), implying that PGPR was not enough to wrap all the water droplets at this time, and the W/O emulsion showed poor stability. When the gelatin concentration reached 4.0%, the W/O emulsion had no water layer, and the emulsion would not flow when standing upside down (Figure 1A), showing the highest stability. This may be related to the interface adsorption of PGPR and the network in the oil phase generated by interface adsorption [27]. On the other hand, with the increase of gelatin concentration, the gel network structure would be formed in the internal aqueous solution due to the good gel characteristics of gelatin. The gel water droplets could reduce the flow and collision of water droplets, and inhibit the coalescence, aqueous precipitation, and fat floating of W/O emulsions, thus greatly enhancing the stability of W/O emulsions [28].

### 3.2. Analysis of Interfacial Tension

**Figure 2:**
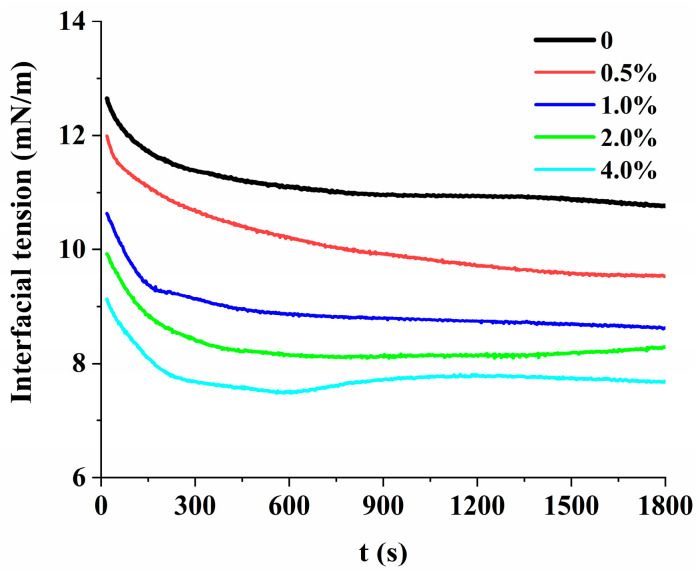
The interfacial tension of PGPR and gelatin (0, 0.5%, 1.0%, 2.0%, and 4.0%).

The effect of different concentrations of gelatin solution (0, 0.5%, 1.0%, 2.0%, and 4.0%) on the interfacial tension of PGPR is exhibited in Figure 2. The interfacial tension of all samples decreased when the time increased (0~1800 s), which indicated that the PGPR was gradually adsorbed on the surface of water droplets and promoted the production of W/O emulsions [29]. Interestingly, as the gelatin concentration increased (0~4.0%), the affinity with PGPR in the oil phase was enhanced, resulting in decreased interfacial tension between PGPR and gelatin at any time. It is reported that PGPR has advantages over protein in reducing oil-water interfacial tension, and additional protein addition may be more conducive to reducing the interfacial tension, thus enhancing the stability of emulsions by enhancing the accumulation between droplets [18,30]. The decrease of interfacial tension was more conducive to the adsorption of PGPR on the surface of water droplets and enhanced the accumulation of droplets to promote the formation of a gel network, thus improving the stability of W/O emulsions by enhancing the overall network. At 1800 s, the interfacial tension between PGPR and gelatin showed a small value with the increase in gelatin concentration, which implied that the stability of the interfacial film formed by PGPR increased. The reason might be that the interfacial adsorption of PGPR was promoted, which enhanced the interaction between interfacial particles (PGPR) and interfacial particles (PGPR), as well as between interfacial particles (PGPR) and gelatin in the aqueous phase, thus enhancing the stability of the interfacial film and W/O emulsions [7,31]. The results of interfacial tension explained the reasons for the enhancement of stability and WHC in Figure 1.

### 3.3. Analysis of Microscopic and Droplet Size

**Figure 3:**
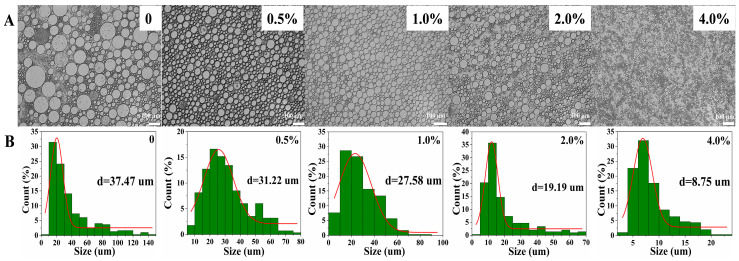
The microscopic (**A**) and droplet size (**B**) of W/O emulsions stabilized by PGPR and gelatin (0, 0.5%, 1.0%, 2.0%, and 4.0%).

The effect of different concentrations of gelatin solution (0, 0.5%, 1.0%, 2.0%, and 4.0%) on the microscopic and droplet size is shown in Figure 3. Clearly, when the W/O emulsion was prepared without introducing gelatin, the emulsion contained many large droplets with a diameter of more than 100 μm, and the droplets were very uneven. With the gelatin concentration in the aqueous phase increased (0~4.0%), the number of large droplets decreased, and the droplets showed a more uniform and decreasing trend, especially 4.0%. The reason is that the addition of gelatin promoted a decrease in the interfacial tension of PGPR, allowing more lipophilic emulsifiers (PGPR) to encapsulate water droplets, resulting in more uniform droplets and a reduction in droplet size [32]. Furthermore, it is worth noting that smaller droplets were more conducive to reducing collisions between droplets, and aqueous gelation can also reduce the flow and collision of water droplets, thus limiting the flow, coalescence, aqueous precipitation, and fat floating of W/O emulsions ultimately greatly enhancing the stability of W/O emulsions [33]. In order to more accurately represent the droplet size, about 300 droplets on the microscopic map were selected randomly to calculate the droplet distribution and average diameter of W/O emulsions, and the results are shown in Figure 3B. For the control group (0), the number of large droplets (≥80 μm) was 12.71%, and the treated groups almost did not contain large droplets (≥80 μm). As the gelatin concentration increased (0~4.0%), the main distribution peak of droplet distribution shifted from 20~25 μm to 7.5 μm, and the average diameter shifted from 37.47 μm to 7.5 μm, indicating that the addition of gelatin in the aqueous phase reduced the droplet diameter and increased the W/O emulsion stability, which is consistent with both intuitive (Figure 1A) and microscopic results.

### 3.4. Analysis of Viscosity

**Figure 4:**
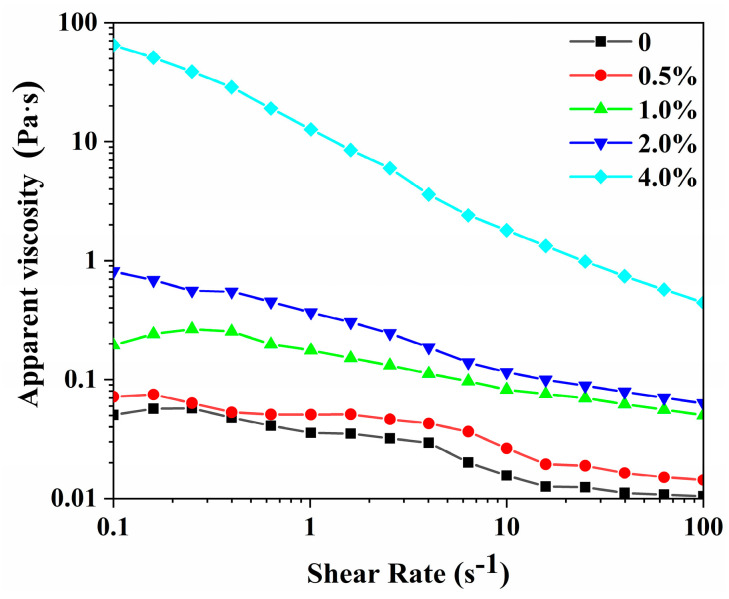
The viscosity of W/O emulsions stabilized by PGPR and gelatin (0, 0.5%, 1.0%, 2.0%, and 4.0%).

The effect of different concentrations of gelatin solution (0, 0.5%, 1.0%, 2.0%, and 4.0%) on the viscosity of W/O emulsions is revealed in Figure 4. The apparent viscosity of all the W/O emulsions decreased with the increase in shear rate (0.1~100 s^−1^), showing the property of shear thinning and indicating that the W/O emulsion belonged to pseudoplastic fluids [34]. With the gelatin concentration in the aqueous phase increased (0~4.0%), the viscosity of W/O emulsions increased obviously at any shear rate. On the one hand, the increased adsorption of PGPR promoted the accumulation of droplets and enhanced the network structure of the W/O emulsion by enhancing the interaction with gelatin in the water phase. On the other hand, the gelation of the water phase also promoted the formation of tight structures, thus improving the apparent viscosity and stability of the W/O emulsion [35]. Moreover, at lower gelatin concentrations (0~2.0%), the viscosity of W/O emulsions was less than 1 Pa s. The addition of gelatin may mainly improve the interfacial stability of W/O emulsions at this time. At 4.0% gelatin concentration, the viscosity increased, which may be mainly due to the formation of a tight overall network structure through the accumulation of droplets, the enhanced interaction between the interface and the aqueous phase, and the gelation of the aqueous phase. The increase in viscosity is more conducive to limiting the movement and aggregation of water droplets and improving the stability of W/O emulsions, consistent with Figure 1A.

### 3.5. Analysis of Storage Stability

**Figure 5:**
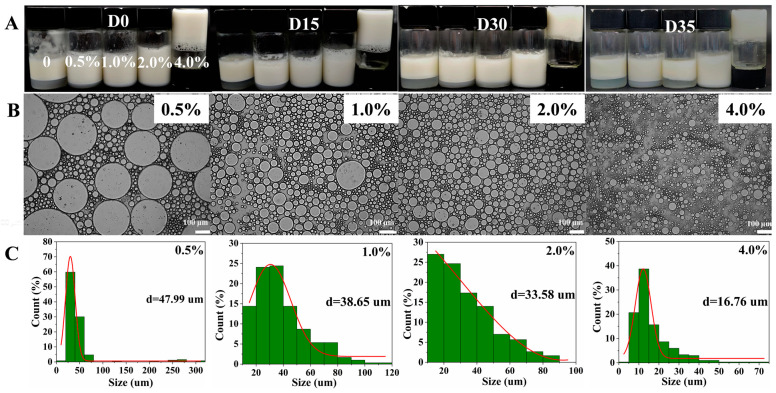
The storage stability of W/O emulsions stabilized by PGPR and gelatin (0, 0.5%, 1.0%, 2.0%, and 4.0%); (**A**) appearance, (**B**) microscopic, (**C**) droplet size.

The effect of different concentrations of gelatin solution (0, 0.5%, 1.0%, 2.0%, and 4.0%) on the storage stability of W/O emulsions is revealed in Figure 5. From the visual graph (Figure 5A), it can be seen that all samples showed almost no obvious changes after 15 d and 30 d of storage, indicating that W/O emulsions showed good storage stability. After 35 d of storage, the W/O emulsion prepared without introducing gelatin showed instability, such as fat floating (as exhibited by the red arrow). While all treatment groups showed no obvious change in comparison with fresh emulsions, they displayed extremely high stability, especially 4.0%, which is related to the tight overall network of W/O emulsions [36,37]. Due to the fat floating of W/O emulsions stabilized by only PGPR, the microcosmic and average diameter of W/O emulsions in the treatment group were tested. The microscopic results (Figure 5B) showed that the droplet size of W/O emulsions gradually decreased with the increase of gelatin concentration and showed a more uniform state. The average diameter results (Figure 5C) showed that the average diameter of W/O emulsions decreased from 47.99 μm to 16.76 μm. Compared with fresh W/O emulsions (Figure 1A), the average diameter of emulsions after 35 d of storage showed a certain increase, which may be caused by aggregation and flocculation [38].

### 3.6. Analysis of Thermal Stability

**Figure 6:**
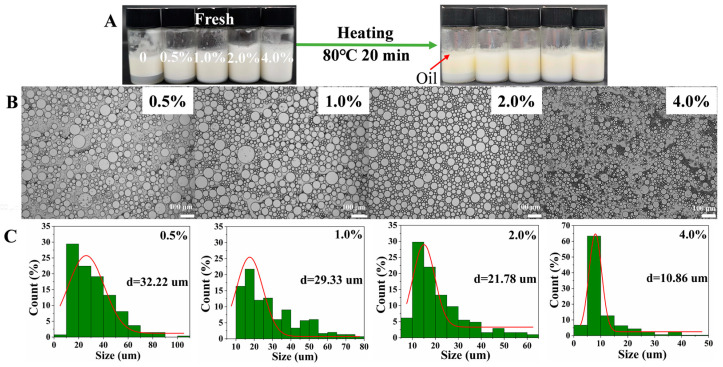
The thermal stability of W/O emulsions stabilized by PGPR and gelatin (0, 0.5%, 1.0%, 2.0%, and 4.0%); (**A**) appearance, (**B**) microscopic, (**C**) droplet size.

The effect of different concentrations of gelatin solution (0, 0.5%, 1.0%, 2.0%, and 4.0%) on the thermal stability of W/O emulsions is revealed in Figure 6. After the W/O emulsion was heated (20 min at 80 °C), the control group (0) showed fat floating (as exhibited by the red arrow), showed poor thermal stability. Compared with the control group (0), the water layer in the treatment group (0.5~2.0%) slightly increased. While the W/O emulsion in 4.0% did not appear to have water and oil separation, it indicated that the structure of the W/O emulsion remained relatively complete and showed high thermal stability. Similarly, the microscopic and average diameter showed that the W/O emulsion formed relatively large droplets in comparison with the fresh emulsion (Figure 1A), showing a relative non-uniformity, the average diameter showed a slight increase (~2 μm), indicating that the addition of gelatin improved the thermal stability of the W/O emulsion to a certain extent by enhancing the overall network.

### 3.7. Analysis of Freeze–Thaw Stability

**Figure 7:**
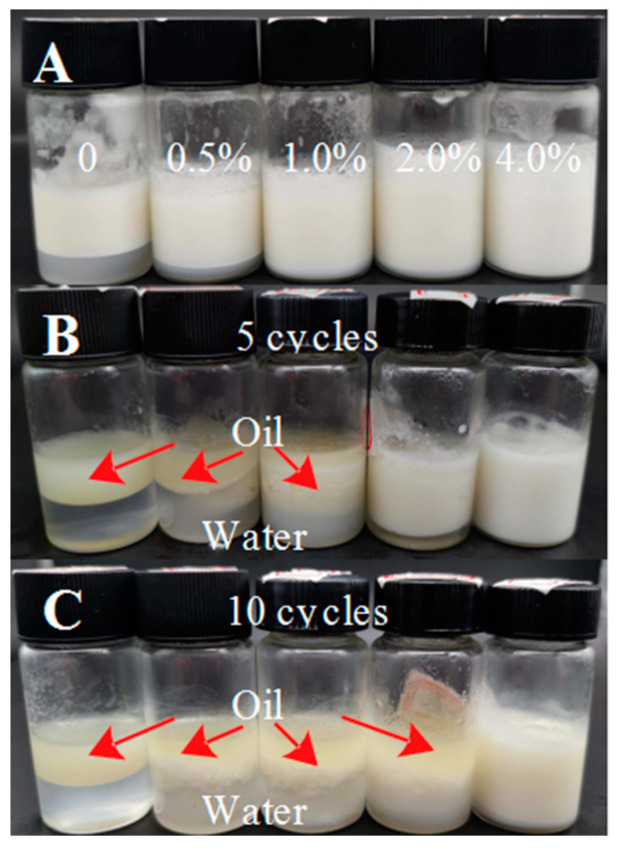
The freeze–thaw stability (appearance) of W/O emulsions stabilized by PGPR and gelatin (0, 0.5%, 1.0%, 2.0%, and 4.0%); (**A**) fresh emulsions, **(B**) emulsions after 5 freeze–thaw cycles, (**C**) emulsions after 10 freeze–thaw cycles.

The effect of different concentrations of gelatin solution (0, 0.5%, 1.0%, 2.0%, and 4.0%) on the freeze–thaw stability of W/O emulsions is revealed in Figure 7. After five freeze–thaw cycles, the W/O emulsion produced a large amount of fat floating (as exhibited by the red arrow). When the gelatin concentration increased to 2.0%, the W/O emulsion did not produce fat floating but produced aqueous precipitation. Only at 4.0% gelatin concentration, there is almost no obvious change in W/O emulsions, and the structure of emulsions remained relatively good. After ten freeze–thaw cycles, the structure of all W/O emulsions was destroyed, and only the W/O emulsion stabilized by PGPR and 4.0% gelatin remained stable, indicating that the induced aqueous phase gelation can improve the freeze–thaw stability of W/O emulsions to a certain extent, of which the W/O emulsion has the best freeze–thaw stability at 4.0% gelatin. The freezing point of soybean oil is about −10 °C, lower than the freezing point of the aqueous phase, and the aqueous phase crystallized before the oil phase. During the freezing process, the gradually formed ice crystals forced droplets into the unfrozen oil phase, destroyed the interfacial film formed by PGPR, the interaction between PGPR and gelatin in the aqueous phase, and the network structure in the oil phase, resulting in a large amount of aqueous phase being separated and fat floating. At 4.0% gelatin concentration, the aqueous gel strength and the interaction between droplets were so strong that they formed a compact overall network, which is more conducive to resisting the destruction of ice crystals and the fat floating, thus showed relatively good freeze–thaw stability [39,40,41].

## 4. Conclusions

This work proposed the strategy of inducing aqueous gelation to improve the stability of W/O emulsions with the medium aqueous phase and mainly evaluated the influence of different gelatin concentrations on the characteristics of W/O emulsions. Results exhibited that the WHC of emulsions was increased significantly (33.50~6.32%, *p* < 0.05) with the gelatin concentration increased, and the emulsion remained as a W/O emulsion. The interface and microscopic results showed that the interfacial tension between PGPR and gelatin decreased (13.34~9.48 mN/m, 10.76~7.69 mN/m) as the gelatin concentration increased, which promoted the adsorption of PGPR at the oil-water interface, allowed more PGPR particles to wrap around water droplets, thereby reduced the non-uniformity and particle size of the droplets. The strengthened interfacial adsorption enhanced the interaction between the interfacial particles (PGPR) as well as the interfacial particles (PGPR) and gelatin in the aqueous phase, which further enhanced the stability of the interface and the strength of the network in the oil phase. Moreover, the increased gelatin concentration was conducive to the induction of aqueous gelation, thus forming a tight overall network through the aqueous gelation and the accumulation of droplets in the oil phase. The enhancement of the overall network could improve the storage stability, thermal stability, and freeze–thaw stability, especially at 4.0% gelatin concentration.

## Data Availability

The data are available upon request from the corresponding author.

## References

[1] Liu F.G., Liang X.P., Yan J., Zhao S.L., Li S.Q., Liu X.B., Ngai T., McClements D.J. (2022). Tailoring the properties of double-crosslinked emulsion gels using structural design principles: Physical characteristics, stability, and delivery of lycopene. Biomaterials.

[2] Xia J., Sun X., Jia P., Li L., Xu K., Cao Y., Lü X., Wang L. (2023). Multifunctional sustainable films of bacterial cellulose nanocrystal-based, three-phase pickering nanoemulsions: A promising active food packaging for cheese. Chem. Eng. J..

[3] He X., Liu J., Li Z., Samchek M., Gates I., Hu J., Lu Q. (2022). Aqueous condition-tolerated high internal phase oil-in-water Pickering emulsion as building block for engineering 3D functional materials. Chem. Eng. J..

[4] Liu Q., Zhai W. (2022). Hierarchical Porous Ceramics with Distinctive Microstructures by Emulsion-Based Direct Ink Writing. ACS Appl. Mater. Interface.

[5] Zhang C., Gao Y., Wu Y., Zheng Z., Xie Y., Li Y., Li B., Pei Y., Liu S. (2023). Construction of stable O/W/O multiple emulsions using beeswax to control the melting point of the continuous oil phase. Food Hydrocoll..

[6] Tenorio-Garcia E., Araiza-Calahorra A., Simone E., Sarkar A. (2022). Recent advances in design and stability of double emulsions: Trends in Pickering stabilization. Food Hydrocoll..

[7] Gu X., Du L., Meng Z. (2023). Comparative study of natural wax-based W/O emulsion gels: Microstructure and macroscopic properties. Food Res. Int..

[8] Li X., Meng R., Xu B., Zhang B., Cui B., Wu Z. (2022). Function emulsion gels prepared with carrageenan and zein/carboxymethyl dextrin stabilized emulsion as a new fat replacer in sausages. Food Chem..

[9] Song J., Sun Y., Wang H., Tan M. (2023). Preparation of high internal phase Pickering emulsion by centrifugation for 3D printing and astaxanthin delivery. Food Hydrocoll..

[10] Liu Y., Huang Y., Wang Y., Zhong J., Li S., Zhu B., Dong X. (2023). Application of cod protein-stabilized and casein-stabilized high internal phase emulsions as novel fat substitutes in fish cake. LWT.

[11] Zhang L., Zaky A.A., Zhou C., Chen Y., Su W., Wang H., Abd El-Aty A.M., Tan M. (2022). High internal phase Pickering emulsion stabilized by sea bass protein microgel particles: Food 3D printing application. Food Hydrocoll..

[12] Gao Y., Lei Y., Wu Y., Liang H., Li J., Pei Y., Li Y., Li B., Luo X., Liu S. (2021). Beeswax: A potential self-emulsifying agent for the construction of thermal-sensitive food W/O emulsion. Food Chem..

[13] Li J., Guo C., Cai S., Yi J., Zhou L. (2023). Fabrication of anthocyanin-rich W1/O/W2 emulsion gels based on pectin–GDL complexes: 3D printing performance. Food Res. Int..

[14] Yu H., Wang H., Su W., Song Y., Zaky A.A., Abd El-Aty A.M., Tan M. (2022). Co-delivery of hydrophobic astaxanthin and hydrophilic phycocyanin by a pH-sensitive water-in-oil-in-water double emulsion-filled gellan gum hydrogel. Food Hydrocoll..

[15] Wang F., Guo L., Liu H., Tao H., Yu B., Zhao H., Li J., Tao H., Cui B., Wang Y. (2023). Water-in-oil oleogel with biphasic stabilization for fabrication of low-fat salad dressing. Food Hydrocoll..

[16] Zhu Q., Qiu S., Zhang H., Cheng Y., Yin L. (2018). Physical stability, microstructure and micro-rheological properties of water-in-oil-in-water (W/O/W) emulsions stabilized by porcine gelatin. Food Chem..

[17] Chen M., Li W., Wang W., Cao Y., Lan Y., Huang Q., Xiao J. (2022). Effects of gelation on the stability, tribological properties and time-delayed release profile of double emulsions. Food Hydrocoll..

[18] Zhang M., Fan L., Liu Y., Li J. (2022). A mechanistic investigation of the effect of dispersion phase protein type on the physicochemical stability of water–in–oil emulsions. Food Res. Int..

[19] Huang H., Belwal T., Aalim H., Li L., Lin X., Liu S., Ma C., Li Q., Zou Y., Luo Z. (2019). Protein-polysaccharide complex coated W/O/W emulsion as secondary microcapsule for hydrophilic arbutin and hydrophobic coumaric acid. Food Chem..

[20] Zhang T., Xu J., Zhang Y., Wang X., Lorenzo J.M., Zhong J. (2020). Gelatins as emulsifiers for oil-in-water emulsions: Extraction, chemical composition, molecular structure, and molecular modification. Trends Food Sci. Technol..

[21] Tang C., Zhou K., Zhu Y., Zhang W., Xie Y., Wang Z., Zhou H., Yang T., Zhang Q., Xu B. (2022). Collagen and its derivatives: From structure and properties to their applications in food industry. Food Hydrocoll..

[22] Feng X., Dai H., Yu Y., Wei Y., Tan H., Tang M., Ma L., Zhang Y. (2022). Adjusting the interfacial property and emulsifying property of cellulose nanofibrils by ultrasonic treatment combined with gelatin addition. Food Hydrocoll..

[23] Feng X., Dai H., Ma L., Yu Y., Tang M., Li Y., Hu W., Liu T., Zhang Y. (2019). Food-Grade Gelatin Nanoparticles: Preparation, Characterization, and Preliminary Application for Stabilizing Pickering Emulsions. Foods.

[24] Liu Y., Lee W.J., Tan C.P., Lai O.M., Wang Y., Qiu C. (2022). W/O high internal phase emulsion featuring by interfacial crystallization of diacylglycerol and different internal compositions. Food Chem..

[25] Balcaen M., Steyls J., Schoeppe A., Nelis V., Van der Meeren P. (2021). Phosphatidylcholine-depleted lecithin: A clean-label low-HLB emulsifier to replace PGPR in w/o and w/o/w emulsions. J. Colloid Interface Sci..

[26] Cheng C., Gao H., McClements D.J., Zeng H., Ma L., Zou L., Miao J., Wu X., Tan J., Liang R. (2022). Impact of polysaccharide mixtures on the formation, stability and EGCG loading of water-in-oil high internal phase emulsions. Food Chem..

[27] García-González D.O., Yánez-Soto B., Dibildox-Alvarado E., Ornelas-Paz J.D.J., Pérez-Martínez J.D. (2021). The effect of interfacial interactions on the rheology of water in oil emulsions oleogelled by candelilla wax and saturated triacylglycerols. LWT.

[28] Tian H., Xiang D., Wang B., Zhang W., Li C. (2021). Using hydrogels in dispersed phase of water-in-oil emulsion for encapsulating tea polyphenols to sustain their release. Colloids Surf. A.

[29] Zhang M., Fan L., Liu Y., Li J. (2023). Effects of alkali treatment on structural and functional properties of chickpea protein isolate and its interaction with gallic acid: To improve the physicochemical stability of water–in–oil emulsions. Food Hydrocoll..

[30] Perez-Moral N., Watt S., Wilde P. (2014). Comparative study of the stability of multiple emulsions containing a gelled or aqueous internal phase. Food Hydrocoll..

[31] Su J., Flanagan J., Hemar Y., Singh H. (2006). Synergistic effects of polyglycerol ester of polyricinoleic acid and sodium caseinate on the stabilisation of water–oil–water emulsions. Food Hydrocoll..

[32] Gülseren İ., Corredig M. (2014). Interactions between polyglycerol polyricinoleate (PGPR) and pectins at the oil–water interface and their influence on the stability of water-in-oil emulsions. Food Hydrocoll..

[33] Gomes A., Costa A.L.R., de Assis Perrechil F., Da Cunha R.L. (2016). Role of the phases composition on the incorporation of gallic acid in O/W and W/O emulsions. J. Food Eng..

[34] Zhang R., Yu J.J., Liu N., Gao Y., Mao L. (2022). W/O emulsions featuring ethylcellulose structuring in the water phase, interface and oil phase for multiple delivery. Carbohyd Polym..

[35] Zhu Q., Pan Y., Jia X., Li J., Zhang M., Yin L. (2019). Review on the Stability Mechanism and Application of Water-in-Oil Emulsions Encapsulating Various Additives. Compr. Rev. Food Sci. Food Saf..

[36] Chevalier R.C., Gomes A., Cunha R.L. (2021). Tailoring W/O emulsions for application as inner phase of W/O/W emulsions: Modulation of the aqueous phase composition. J. Food Eng..

[37] Chevalier R.C., Gomes A., Cunha R.L. (2022). Role of aqueous phase composition and hydrophilic emulsifier type on the stability of W/O/W emulsions. Food Res. Int..

[38] Gao Y., Mao J., Meng Z. (2023). Tracing distribution and interface behavior of water droplets in W/O emulsions with fat crystals. Food Res. Int..

[39] Thanasukarn P., Pongsawatmanit R., McClements D.J. (2004). Influence of emulsifier type on freeze–thaw stability of hydrogenated palm oil-in-water emulsions. Food Hydrocoll..

[40] Luo S., Hu X., Pan L., Zheng Z., Zhao Y., Cao L., Pang M., Hou Z., Jiang S. (2019). Preparation of camellia oil-based W/O emulsions stabilized by tea polyphenol palmitate: Structuring camellia oil as a potential solid fat replacer. Food Chem..

[41] Ghosh S., Rousseau D. (2009). Freeze–thaw stability of water-in-oil emulsions. J. Colloid Interface Sci..

